# Unlocking Zenith Electronic State of Ni Sites in Ligand‐Engineered COFs for Efficient •O_2_^−^‐Driven Uranium Photoreduction

**DOI:** 10.1002/advs.77949

**Published:** 2026-09-27

**Authors:** Guihong Wu, Fengtao Yu, Lin Wen, Sai‐Jin Xiao, Fangru Song, Jian‐Ding Qiu

**Affiliations:** ^1^ National Key Laboratory of Uranium Resources Exploration‐Mining and Nuclear Remote Sensing East China University of Technology Nanchang China

**Keywords:** direct ligand engineering, Ni‐diketimine COFs, photocatalytic uranium reduction, superoxide radical, zenith electronic state

## Abstract

Photocatalytic uranium reduction via the superoxide radical (•O_2_^−^) pathway is a sustainable route, but its efficiency is constrained by rapid charge recombination and poor O_2_ activation. Herein, we develop a ligand engineering strategy to construct a series of Ni‐diketimine‐linked COFs by varying the diketone units directly coordinated to the Ni centers, enabling precise tuning of the Ni electronic state. The acenaphthylene‐1,2‐dione‐derived Ni‐COF‐E reaches a peak optimum within the present series, termed the “Zenith electronic state”, as evidenced by the highest ligand‐to‐metal charge transfer (0.584 e) and the largest charge transfer distance (1.700 Å) among the three Ni‐COFs in the *S*_1_ excited state. This zenith electron density balances efficient photogenerated electron capture with enhanced O_2_ adsorption and a lowered barrier for •O_2_^−^ formation, which is corroborated by combined experimental and theoretical analyses. Consequently, Ni‐COF‐E delivers a record •O_2_^−^ generation rate of 8.48 µM h^−1^ and achieves >99% uranium removal from real wastewater. This work establishes direct ligand engineering as a powerful strategy for tuning single‐metal sites in COFs and positions the Zenith electronic state as a new design principle for •O_2_^−^‐mediated photocatalysis.

## Graphical abstract

We develop a ligand engineering strategy to construct Ni‐diketimine‐linked COFs with precisely tuned Ni electronic states. By varying the coordinated diketone units, we achieve an optimal electronic configuration that enhances O_2_ adsorption and lowers the energy barrier for •O_2_^−^ generation. This synergy enables efficient •O_2_^−^‐mediated photocatalytic reduction of U(VI) from real wastewater.

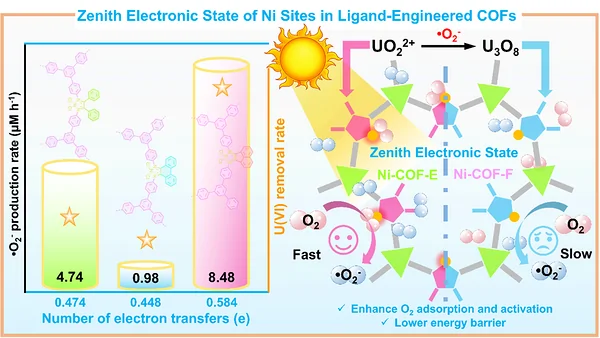

## 1 Introduction

Photocatalytic reduction of uranium from wastewater offers a sustainable route to nuclear fuel using solar energy [1, 2, 3, 4]. In aerated water, this process typically proceeds via an indirect superoxide radical (•O_2_^−^) pathway, wherein photogenerated electrons reduce O_2_ to •O_2_^−^, which then reduces U(VI) to U(IV) [5, 6, 7]. Using O_2_ as an electron shuttle avoids the need for strong uranyl adsorption sites and rapid two‐electron injection required in direct reduction. More critically, the •O_2_^−^‐mediated pathway tolerates complex wastewater matrices where competing ions or organics often poison direct reduction sites, because •O_2_^−^ forms in situ from ubiquitous dissolved O_2_ without relying on pre‐adsorbed uranium [8, 9, 10, 11]. Nevertheless, existing photocatalysts suffer from insufficient •O_2_^−^ production, a problem stemming from rapid charge recombination that depletes photogenerated electrons and sluggish O_2_ adsorption/activation kinetics that hinder •O_2_^−^ formation at catalytic sites. Therefore, developing advanced materials for efficient •O_2_^−^ generation is urgently needed [12, 13].

Covalent organic frameworks (COFs) have emerged as promising platforms, offering structural designability for the precise integration of functional sites [14, 15, 16, 17, 18]. Nevertheless, purely organic COFs still face two intertwined limitations, namely inefficient charge separation with slow electron transfer and inadequate capability for O_2_ binding and activation [19, 20, 21, 22, 23]. More importantly, in purely organic COFs the sites of electron accumulation (acceptor units) are rarely the same as the O_2_ adsorption/activation sites, creating a spatial mismatch. This forces photogenerated electrons to diffuse over long distances to reach catalytic centers, a process that competes with sub‐nanosecond charge recombination and severely limits •O_2_^−^ yields.

**SCHEME 1:**
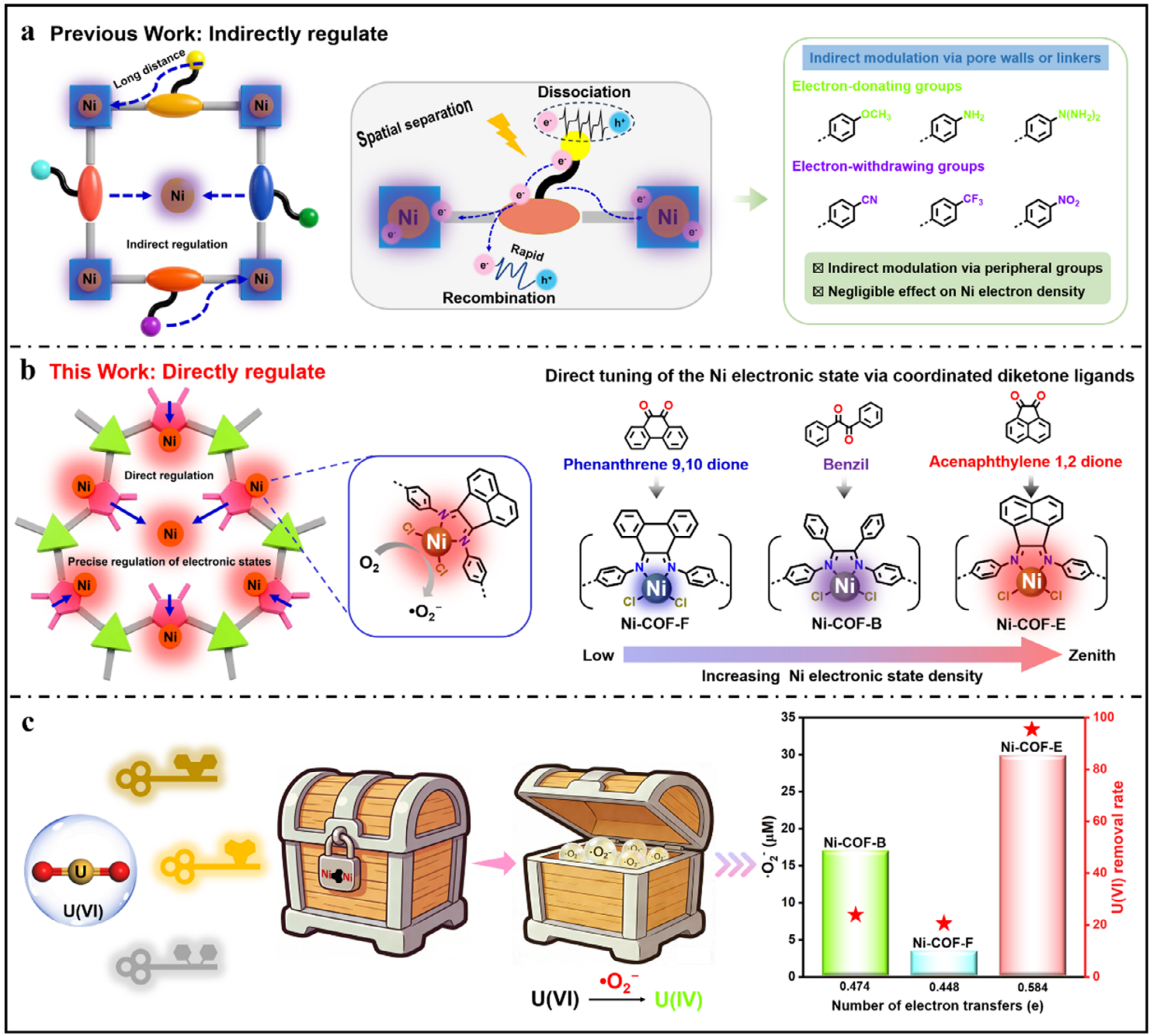
(a) Indirect regulation of the Ni sites' electronic state in previous work. (b) Direct regulation of the Ni sites' electronic state in this work. (c) The zenith electronic state effectively enhances the production of •O_2_^−^.

To overcome these limitations, incorporating Ni sites into COFs has proven effective. Ni sites act as electron traps to promote charge separation and migration, while also providing dedicated sites for O_2_ adsorption and activation via d‐π* back donation, thereby substantially boosting •O_2_^−^ production and achieving the goal of eliminating the spatial mismatch between electron accumulation and oxygen binding [24, 25, 26]. Consequently, the •O_2_^−^ yield is largely dictated by O_2_ adsorption and activation at the Ni sites, which in turn is fundamentally governed by the local electronic state of the Ni center [24, 27, 28, 29, 30]. Previous strategies have attempted to modulate this electronic state indirectly, for example by introducing electron‐donating or electron‐withdrawing groups onto the pore walls or linkers (Scheme 1a). However, because of the spatial separation between these peripheral functional groups and the catalytic metal center, their influence on Ni electron density is negligible, resulting in limited control over the catalytic microenvironment [31, 32, 33, 34].

To break this limitation, we adopt a direct ligand engineering strategy that involves varying the diketone ligands directly coordinated to the Ni center, thereby precisely tuning the Ni electronic state without the inefficiencies of remote functionalization (Scheme 1b). By systematically varying the coordinated diketone units, we discover that the Ni electronic state does not change monotonically; rather, it reaches a peak optimum within the present series when using the acenaphthylene‐1,2‐dione‐derived ligand. We term this peak optimum the “Zenith electronic state”, which is characterized by the highest ligand‐to‐metal charge transfer in the *S*_1_ state, as revealed by TD‐DFT calculations. This summit electron density strikes a delicate balance, enabling strong O_2_ adsorption while minimizing the kinetic barrier for •O_2_^−^ generation. To our knowledge, this is the first demonstration that direct coordination tuning can drive a single‐metal site into a defined Zenith regime, which thus serves as a new descriptor for •O_2_^−^‐mediated photocatalysis.

As a proof of concept, we report a series of Ni‐diketimine‐linked COFs (Ni‐COF‐B, Ni‐COF‐F, and Ni‐COF‐E) constructed from three diketone ligands (benzil, phenanthrene‐9,10‐dione, and acenaphthylene‐1,2‐dione). Ni‐COF‐E, derived from acenaphthylene‐1,2‐dione, achieves the Zenith electronic state. Under light irradiation, it transfers photogenerated electrons to Ni sites, delivering a record •O_2_^−^ generation rate of 8.48 µM h^−1^ and achieving over 99% uranium removal from real wastewater (Scheme 1c). Comprehensive experimental and theoretical studies reveal that the Zenith Ni sites optimize O_2_ adsorption and lower the •O_2_^−^ formation barrier. Our work establishes direct ligand engineering as a powerful strategy to tune single‐metal sites in COFs, and positions the Zenith electronic state as a new design concept for high‐performance •O_2_^−^‐mediated photocatalysts beyond uranium extraction.

## 2 Results and Discussion

### 2.1 Synthesis and Characterization of COFs

**FIGURE 1:**
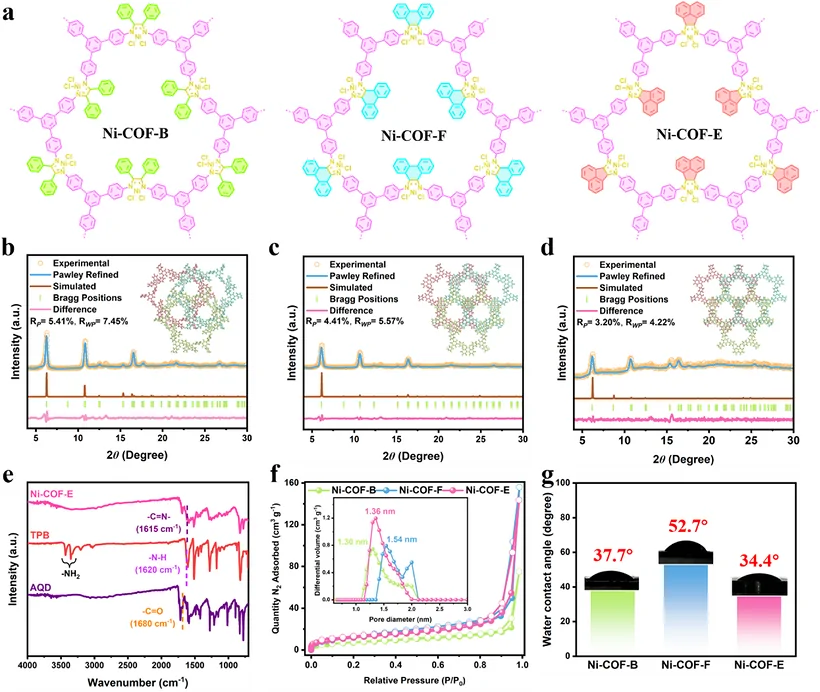
(a) Design of Ni‐COFs with varied ligands to tune the electronic states of Ni sites. (b‐d) PXRD patterns with insets showing the ABC‐stacked structural models of the corresponding Ni‐COFs. (e) FT‐IR spectra. (f) Nitrogen adsorption‐desorption isotherms and pore size distribution (inset). (g) Water contact angle measurements.

The three Ni‐COFs (Figure 1a) were synthesized via a one‐pot condensation reaction. 1,3,5‐tris(4‐aminophenyl)benzene (TPB) was condensed with three different diketones (benzil, phenanthrene‐9,10‐dione, and acenaphthylene‐1,2‐dione) in the presence of NiCl_2_·6H_2_O under solvothermal conditions following a previously reported single‐site coordination strategy (see Supporting Information for details) [35, 36, 37, 38]. Crystalline structures were first confirmed by powder X‐ray diffraction (PXRD). As shown in Figure 1b–d, all materials exhibited intense and sharp diffraction peaks, indicative of long‐range order [39, 40]. Pawley refinement against the ABC‐stacked structural models converged with low residuals. For Ni‐COF‐B, *R_p_* = 5.41% and *R_wp_* = 7.45%; for Ni‐COF‐F, *R_p_* = 4.41% and *R_wp_* = 5.57%; for Ni‐COF‐E, *R_p_* = 3.20% and *R_wp_* = 4.22%. These results confirmed excellent agreement between experimental data and the simulated patterns. Refined unit‐cell parameters were consistent with the incorporation of the respective Ni‐diketimine linkages derived from the three diketone ligands (Figure S1). Chemical formation of the diketimine linkages and Ni coordination was verified by Fourier‐transform infrared (FT‐IR) spectroscopy. Taking Ni‐COF‐E as a representative (Figure 1e), the characteristic C═O stretch of acenaphthylene‐1,2‐dione at 1680 cm^−1^ disappeared after reaction [41, 42], and the N─H stretches of TPB (3300–3500 cm^−1^) were also absent. Meanwhile, a new intense band appeared at 1615 cm^−1^, assigned to the C═N stretch of the coordinated diketimine [43, 44]. Identical spectral changes were observed for Ni‐COF‐B and Ni─COF─F (Figures S2 and S3).

Morphologies and elemental distributions of the three COFs were examined by scanning electron microscopy (SEM) combined with energy‐dispersive X‐ray (EDX) spectroscopy (Figures S4–S6). Ni‐COF‐B and Ni‐COF‐E exhibited irregular lamellar morphologies, whereas Ni‐COF‐F formed uniform microspheres (0.5–2 µm), likely due to the enhanced interlayer π–π stacking from its larger rigid conjugated plane [45, 46]. Corresponding EDX elemental mapping confirmed the homogeneous distribution of C, N, Cl, and Ni across the entire particle for all Ni‐COFs. No elemental segregation or aggregation was observed, confirming the successful integration of Ni sites into the COF backbone and excluding the presence of aggregated NiCl_2_ species as separate phases. Thus, the Ni sites remain atomically dispersed irrespective of the morphological variations. Nickel content in the as‐synthesized Ni‐COFs was quantified by inductively coupled plasma‐mass spectrometry (ICP‐MS). The measured Ni loadings range from 11.22 to 12.23 wt.%, in excellent agreement with the theoretical values calculated for fully coordinated structures (Figure S7; Table S1). This result confirmed that nearly all diketimine units in the frameworks were coordinated with Ni(II) chloride.

Porosity was revealed by N_2_ adsorption measurements (Figure 1f). Brunauer‐Emmett‐Teller (BET) specific surface areas were calculated to be 26.39, 44.73, and 42.60 m^2^ g^−1^ for Ni‐COF‐B, Ni‐COF‐F, and Ni‐COF‐E, respectively (Figure 1f; Figures S8–S10). Narrow pore‐size distribution peaks were centered at 1.30, 1.54, and 1.36 nm, respectively, closely matching the simulated channel dimensions of the ABC‐stacked models (Figure S11). Surface wettability, a key factor for aqueous‐phase photocatalysis, was quantified by water contact‐angle measurements (Figure 1g). Ni‐COF‐E exhibited the smallest contact angle (34.4°), indicating superior hydrophilicity relative to Ni‐COF‐B (37.7°) and Ni‐COF‐F (52.7°), which enhanced the accessibility of both Ni sites and dissolved O_2_ in the reaction medium [47]. Finally, all three Ni‐COFs exhibited thermal decomposition temperatures above 400°C in thermogravimetric analysis (TGA, Figure S12), ensuring their structural integrity and providing a robust platform for subsequent photocatalytic uranium extraction.

Collectively, the above characterizations demonstrated that a series of isostructural Ni‐COFs with distinct diketone ligands have been successfully synthesized. Their high crystallinity, uniform Ni dispersion, and suitable hydrophilicity set the stage for investigating how the Ni electronic state influences photocatalytic performance.

### 2.2 Electronic Structures and Charge Separation Performance

**FIGURE 2:**
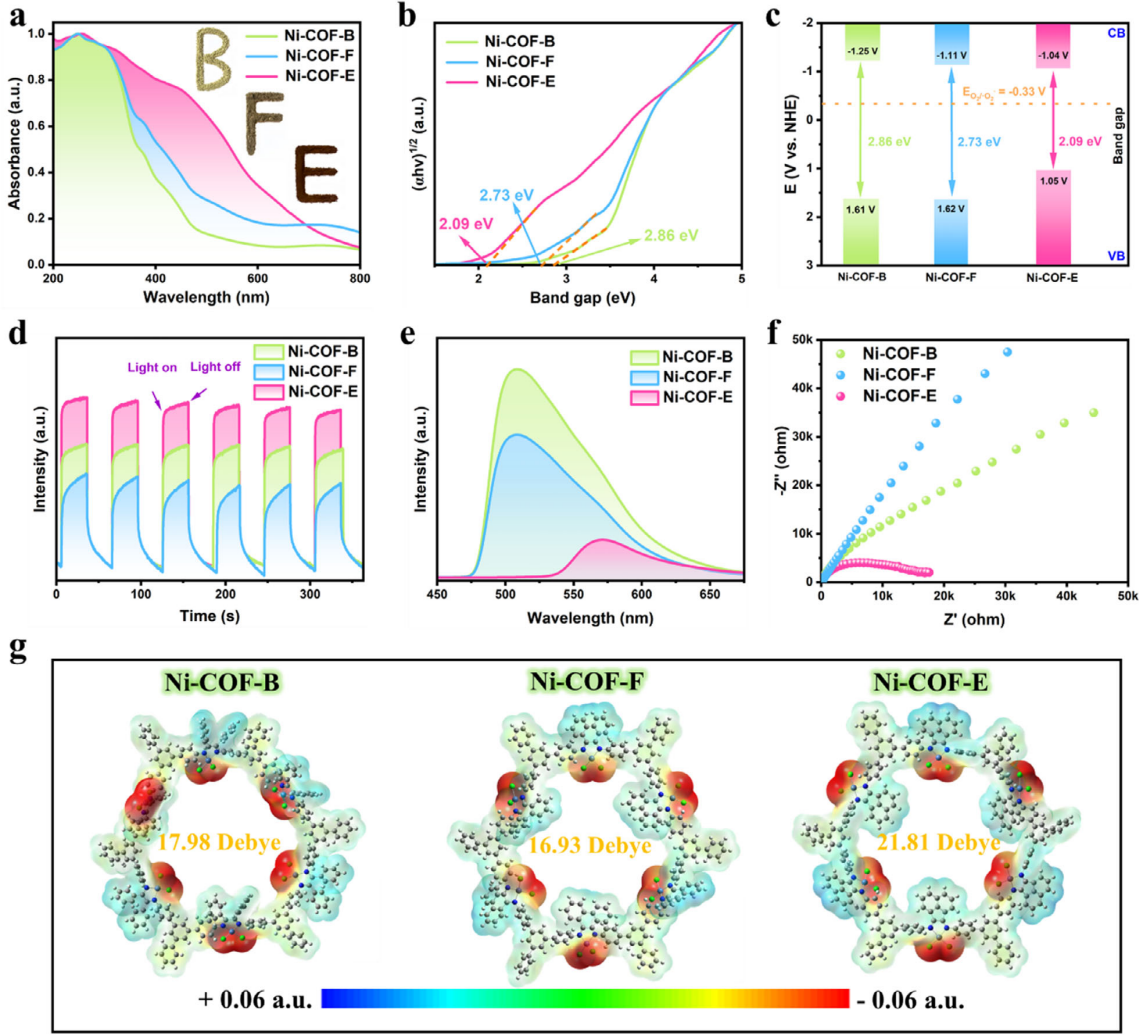
(a) Solid‐state UV–Vis diffuse reflection spectra and apparent color (inset). (b) The bandgaps calculated by Kubelka−Munk theory. (c) Energy band diagram. (d) Transient current responses to on‐off cycles of illumination. (e) Steady‐state PL spectra. (f) EIS Nyquist plots at open‐circuit voltage. (g) DFT calculation results of the electrostatic potential surface and corresponding dipole moment (µ: the unit of dipole moment was Debye).

The optical absorption properties of all Ni‐COFs were examined by UV–vis diffuse reflectance spectroscopy (DRS, Figure 2a). Ni‐COF‐E exhibited a markedly red‐shifted absorption onset and a stronger visible‐light response than Ni‐COF‐B and Ni‐COF‐F. This behavior originated from the acenaphthylene‐1,2‐dione‐derived diketimine ligand, which induced the strongest intramolecular D‐A character and the most effective π‐delocalization within the Ni‐coordinated framework. Corresponding Tauc plots (Figure 2b) gave optical band gaps (*E*_g_) of 2.86, 2.73, and 2.09 eV for Ni‐COF‐B, Ni‐COF‐F, and Ni‐COF‐E, respectively. Band alignment was determined from Mott‐Schottky measurements (Figure S13). The flat‐band potentials were −1.25, −1.11, and −1.04 V (vs. Ag/AgCl) for Ni‐COF‐B, Ni‐COF‐F, and Ni‐COF‐E, respectively [48]. Converting to the normal hydrogen electrode (NHE) scale (E vs. NHE = E vs. Ag/AgCl + 0.2 V) and taking the flat‐band potential as approximately 0.2 V above the conduction band (CB) for *n*‐type semiconductors, the CB values were numerically −1.25, −1.11, and −1.04 V (vs. NHE), respectively. Combining these values with the *E_g_* yielded valence band (VB) positions of +1.61 V (Ni‐COF‐B), +1.62 V (Ni‐COF‐F), and +1.05 V (Ni‐COF‐E) vs. NHE (Figure 2c). Importantly, all Ni‐COFs possessed sufficiently negative CB to thermodynamically drive the reduction of O_2_ to •O_2_^−^ (−0.33 V vs. NHE).

Charge separation and transfer efficiencies were evaluated electrochemically. Under intermittent visible‐light irradiation, Ni‐COF‐E delivered the highest transient photocurrent density (Figure 2d), indicating superior photogenerated carrier generation and separation. Steady‐state photoluminescence (PL) experiments showed that Ni‐COF‐E exhibited the lowest PL intensity, implying the slowest radiative recombination and thus the most efficient charge separation; conversely, Ni‐COF‐B displayed the highest PL intensity, suggesting faster recombination (Figure 2e). Electrochemical impedance spectroscopy (EIS) further confirmed the trend. The Nyquist plot of Ni‐COF‐E presented the smallest semicircle diameter, corresponding to the lowest interfacial charge‐transfer resistance (Figure 2f).

To understand the origin of these differences, density functional theory (DFT) calculations were performed on simplified Ni‐diketimine structural units. Electrostatic potential (ESP) analysis revealed that Ni‐COF‐E exhibited the largest molecular dipole moment (21.81 Debye) among the series (Figure 2g). Complementary HOMO and LUMO distributions showed enhanced spatial separation of frontier orbitals in the acenaphthylene‐1,2‐dione derived system (Figure S14). These ligand‐induced electronic effects created a stronger local electric field that promoted directional electron migration from the COF backbone to the Ni sites.

### 2.3 TD‐DFT Insights into Photoinduced Charge Transfer and Excitonic Effects

**FIGURE 3:**
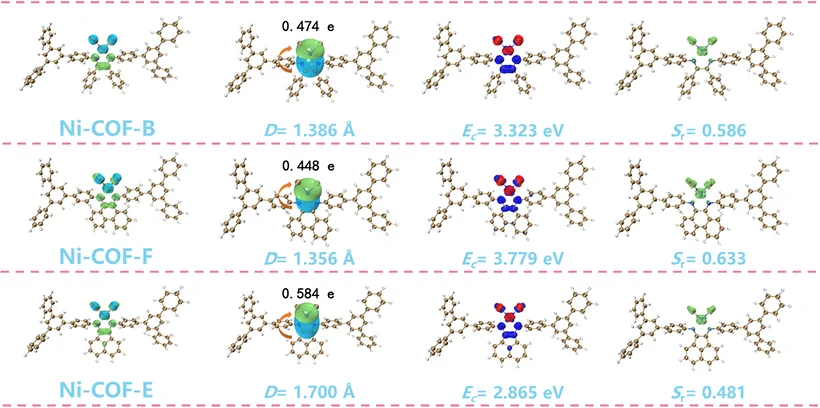
(First column) Electron (cyan) and hole (green) distributions of the S_1_ excited states of Ni‐COFs fragments. (Second column) Smoothed distributions of the electrons and holes. (Third column) Charge density difference between the ground and excited states. (Fourth column) Overlap between the electron and hole distributions. Quantitative charge transfer analysis was performed based on the atomic dipole‐corrected Hirshfeld (ADCH) atomic charges. *D* was denoted as the barycenter distance between the density increment and depletion regions upon electronic excitation. *E_c_* was the Coulomb attractive energy derived from DFT calculations. *S_r_* represented the hole–electron overlap indicator (isovalue = 0.0005 a.u.).

To gain deeper insight into the photoinduced charge transfer dynamics, time‐dependent density functional theory (TD‐DFT) calculations were performed on simplified fragment models of Ni‐COFs. Electron and hole distributions in the first excited state (*S*_1_) were presented in the first column of Figure 3. In all fragments, holes (green) were predominantly localized on the diketimine moiety, whereas electrons (cyan) were concentrated on the Ni center and its immediate coordination environment [49]. The charge density difference maps further revealed that electrons were excited from the diketimine unit (cyan region) and migrated directionally toward the Ni center (green region), confirming efficient intramolecular ligand‐to‐metal charge transfer.

Quantitative charge transfer parameters were calculated to compare the three systems. Among the Ni‐COFs, Ni‐COF‐E exhibited the highest amount of transferred electrons (0.584 e) and the largest charge transfer distance (*D* = 1.700 Å) in the *S*_1_ state (Figure 3, second column). A greater amount of transferred electrons and A larger *D* value and indicated a stronger D‐A interaction. In contrast, Ni‐COF‐B and Ni‐COF‐F showed smaller electron transfer quantities, consistent with their inferior charge separation capabilities. These results confirmed that Ni‐COF‐E possessed the strongest D‐A interaction, enabling superior charge separation. Excitonic effects were evaluated by the Coulomb attractive energy (*E_c_*) and the *S*_r_ index (overlap between electron and hole distributions). For Ni‐COF‐B, *E_c_* = 3.323 eV and *S_r_* = 0.586; for Ni‐COF‐F, *E_c_* = 3.779 eV and *S_r_* = 0.633; for Ni‐COF‐E, *E_c_* = 2.865 eV and *S_r_* = 0.481. Ni‐COF‐E exhibited the lowest *E_c_* and the smallest *S_r_*, indicating a significantly weakened exciton binding. This facilitated rapid dissociation of excitons into free charge carriers upon photoexcitation.

Overall, these TD‐DFT results demonstrated that the acenaphthylene‐1,2‐dione derived ligand in Ni‐COF‐E promoted the most efficient photoinduced charge transfer from the COF backbone to the Ni active site. The well‐separated electron‐hole distribution, the largest charge transfer distance, and the smallest Coulomb attractive energy endowed Ni‐COF‐E with an excellent capability for generating free electrons, which was the prerequisite for efficient •O_2_^−^ production.

Based on these quantitative TD‐DFT analyses, we defined the peak optimum of Ni electronic configuration within the present series as the “Zenith electronic state”, which was characterized by the highest ligand‐to‐metal charge transfer in the *S*_1_ excited state. This definition, grounded in the observed charge transfer parameters, provided a clear and quantifiable criterion for identifying the Zenith state, distinguishing it from a purely descriptive “optimal” state.

### 2.4 Photocatalytic Uranium Extraction Performances

**FIGURE 4:**
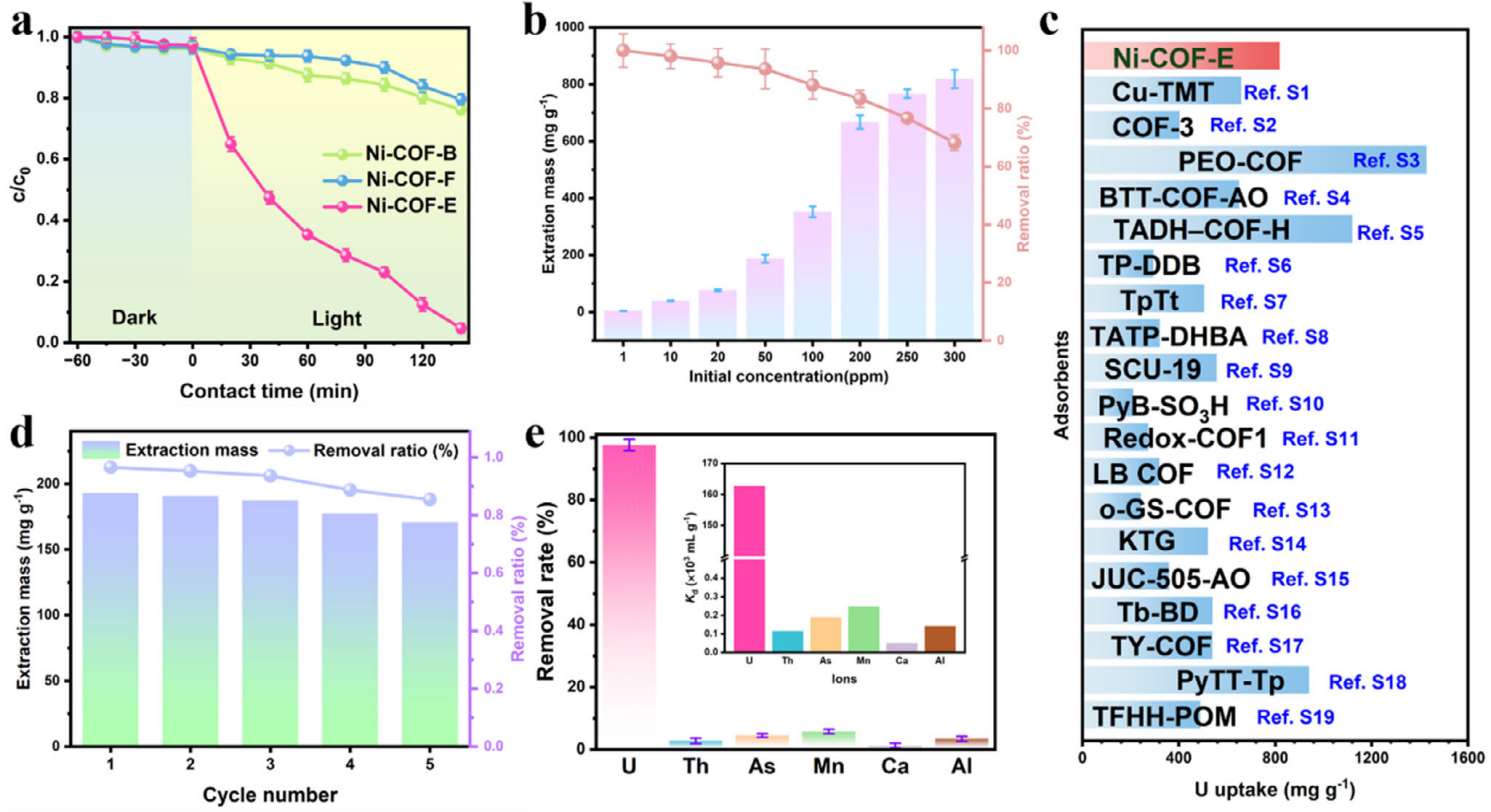
(a) Concentration of U(VI) as a function of time using the obtained Ni‐COFs under dark and irradiated conditions. (b) Removal rate and extracted uranium mass of Ni‐COF‐E as a function of initial U(VI) concentration. (c) Comparison of the uranium extraction capability of Ni‐COF‐E with those of other reported materials. (d) Uranium adsorption capacity and elution efficiency of Ni‐COF‐E. (e) Photocatalytic performance of Ni‐COF‐E for uranium removal from real wastewater (Inset: Competition of coexisting metal ion nitrates or sodium salts, m/V = 0.2 g L^−1^). Error bars represent S.D. n = 3 independent experiments.

The photocatalytic U(VI) removal performances of all Ni‐COFs were systematically evaluated. As shown in Figure 4a, dark adsorption equilibria were established within 60 min, yielding negligible removal efficiencies below 5%, indicating the absence of high‐affinity binding sites for uranyl ions and confirming that subsequent removal under illumination arises from photocatalysis rather than adsorption. Upon light irradiation, Ni‐COF‐E exhibited markedly accelerated reaction kinetics. The U(VI) concentration decreased continuously over 140 min to achieve a removal efficiency of 95.3 %. In contrast, Ni‐COF‐B and Ni‐COF‐F achieved only 23.9% and 20.5% removal, respectively. This striking difference confirmed the superior photoreduction activity of the acenaphthylene‐1,2‐dione‐derived framework [50, 51].

Extraction capacity of Ni‐COF‐E was further quantified across a range of initial U(VI) concentrations (Figure 4b). Ni‐COF‐E delivered a maximum extraction capacity of 818.76 mg g^−1^, substantially outperforming most reported COF‐based photocatalysts and other porous materials (Figure 4c), highlighting the effectiveness of ligand engineering in modulating photoreduction kinetics. Reusability and chemical stability of Ni‐COF‐E were evaluated over five consecutive cycles. As shown in Figure 4d, Ni‐COF‐E retained more than 85% of its initial removal capacity. Given that ICP‐MS showed negligible Ni leaching and the PXRD/FTIR patterns (Figures S15 and S16) of the recovered catalyst remained essentially unchanged, the slight activity loss was attributed to physical loss of catalyst powder during recovery rather than chemical deactivation. Practical applicability of Ni‐COF‐E was validated using real uranium‐containing wastewater (Table S5). Ni‐COF‐E achieved >97% U(VI) removal while exhibiting negligible uptake of competing ions (Figure 4e). Meanwhile, the calculated distribution coefficient (*K*_d_) of uranium was as high as 1.63 × 10^5^ mL g^−1^, significantly higher than that of other competing ions (Figure 4e, inset), indicating that Ni‐COF‐E had a strong affinity and high selectivity for uranium, a finding that underscores its potential for treating genuine nuclear wastewater.

Together, these results demonstrated that the Zenith electronic state of Ni‐COF‐E enabled exceptional photocatalytic U(VI) reduction performance, with high capacity, good reusability, and excellent selectivity in complex wastewater matrices.

### 2.5 The Interaction Between Ni‐COFs and Uranium

**FIGURE 5:**
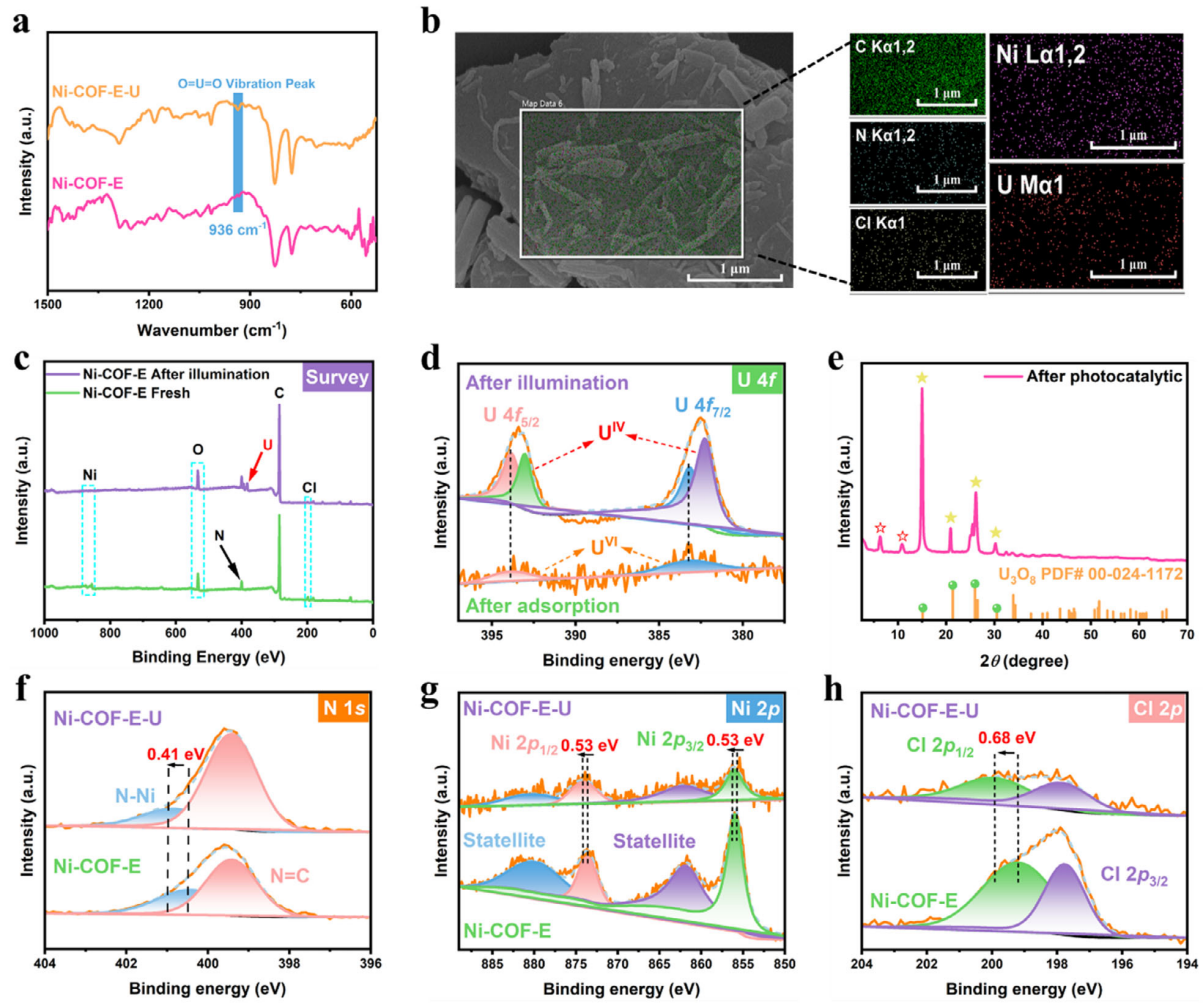
Characterization of pristine Ni‐COF‐E and Ni‐COF‐E‐U (a) FT‐IR spectra. (b) SEM image and the corresponding EDX elemental mappings of Ni‐COF‐E‐U. (c) XPS survey spectra. (d) High‐resolution U 4*f* spectrum (only for Ni‐COF‐E‐U). (e) PXRD pattern of Ni‐COF‐E‐U. (f–h) High‐resolution XPS spectra of N 1*s*, Ni 2*p*, and Cl 2*p*, respectively, for both samples.

To elucidate the interaction between Ni‐COF‐E and uranium species, comprehensive post‐reaction analyses were performed on the used catalyst (denoted as Ni‐COF‐E‐U). Confirmation of uranium attachment was first obtained by FT‐IR spectroscopy. As shown in Figure 5a and Figure S17, Ni‐COF‐E‐U revealed a new absorption band at approximately 936 cm^−1^, absent in pristine Ni‐COF‐E, which was assigned to the asymmetric stretching vibration of the O═U═O bond. SEM and EDX mapping (Figure 5b) revealed that the nanosheet morphology of the pristine COF was largely preserved after the photoreaction, and uranium was uniformly dispersed across the material surface, indicating homogeneous uranium loading.

Valence state change of uranium was probed by X‐ray photoelectron spectroscopy (XPS). Survey spectra (Figure 5c) showed characteristic U 4*f* peaks emerging after illumination. High‐resolution XPS of the U 4*f* region (Figure 5d) provided insight into the photoreduction process. After illumination, two new peaks appeared at 393.03 (U 4*f*_5/2_) and 382.24 eV (U 4*f*_7/2_), corresponding to U(IV), whereas peaks assigned to U(VI) dominated in the pre‐illumination sample. The dominant peak area of the U(IV) species indicated efficient photoreduction of adsorbed U(VI) to U(IV) during photocatalysis. The reduced uranium product was identified by powder X‐ray diffraction (PXRD). As shown in Figure 5e, the diffraction pattern of Ni‐COF‐E‐U matched well with the standard pattern of crystalline U_3_O_8_ (PDF# 00‐024‐1172), definitively identifying the final reduction product [52].

Coordination interaction between Ni‐COF‐E and uranium was further investigated by high‐resolution XPS spectra of key elements. In the N 1*s* region (Figure 5f), the N–Ni peak shifted positively by 0.41 eV compared with the fresh catalyst, indicating a decrease in electron density on the N after illumination. The Ni 2*p* spectrum of fresh Ni‐COF‐E displayed two characteristic peaks at 874.09 (Ni 2*p*_1/2_) and 873.56 eV (Ni 2*p*_3/2_), together with their satellite peaks, assigned to Ni(II). Upon illumination, both the peaks shifted positively by 0.53 eV. Similarly, the Cl 2*p* spectrum (Figure 5h) exhibited a positive shift of 0.68 eV. These systematic binding energy shifts indicated a decrease in electron density on the N, Ni, and Cl atoms, pointing to their participation in the electron transfer process during U(VI) reduction.

Overall, these results demonstrated that under light irradiation, Ni‐COF‐E effectively captured and reduced U(VI) to crystalline U_3_O_8_. The observed shifts in binding energies of N, Ni, and Cl provided direct evidence that these atoms were involved in the electron transfer cascade.

### 2.6 Photocatalytic Mechanism Studies

**FIGURE 6:**
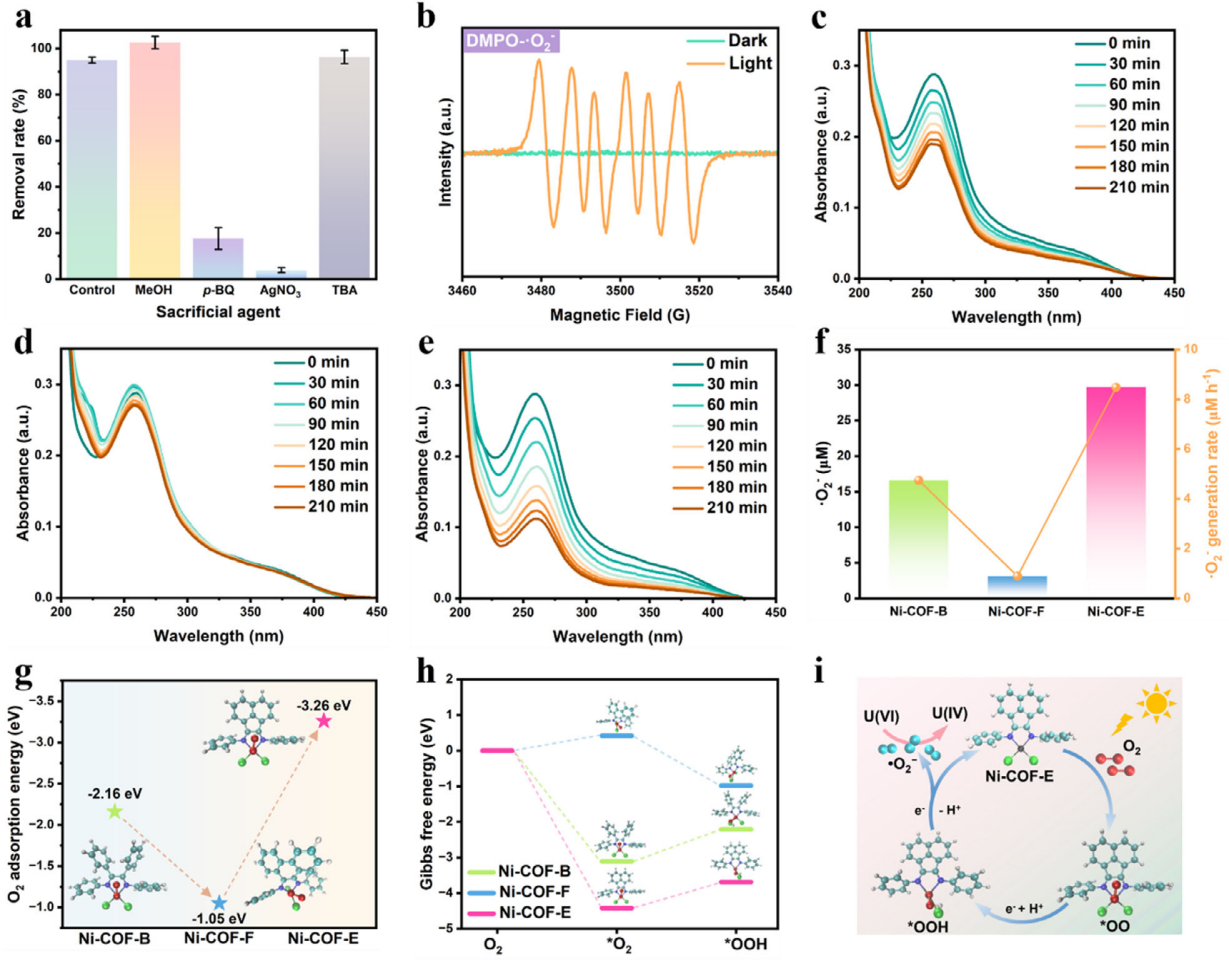
(a) Comparison of the removal rate of uranium by Ni‐COFs‐based photocatalysts under different conditions over 1 h: Control, Methanol, *p*‐BQ, AgNO_3,_ and TBA. Error bars represent S.D. n = 3 independent experiments. (b) EPR spectra of DMPO‐•O_2_^−^. (c‐e) Time‐resolved absorption spectra of NBT of Ni‐COF‐B, Ni‐COF‐F and Ni‐COF‐E, respectively. (f) The photocatalytic yields and rates of •O_2_^−^. (g) The calculated results of the adsorption energy of O_2_ on the catalyst surface. (h) The Gibbs free energy diagram of *O_2_ and *OOH processes for COFs, as well as the corresponding optimized structures. (i) Schematic illustration of the overall •O_2_^−^ production mechanism over Ni‐COF‐E.

To identify the reactive species governing U(VI) photoreduction and the origin of the enhanced activity of Ni‐COF‐E, radical scavenging experiments were conducted (Figure 6a). Addition of methanol (h^+^ scavenger) or tert‐butyl alcohol (TBA, •OH scavenger) caused negligible changes in U(VI) removal efficiency, ruling out the involvement of photogenerated holes and hydroxyl radicals. In contrast, introduction of *p*‐benzoquinone (*p*‐BQ, •O_2_^−^ scavenger) severely suppressed uranium reduction, dropping the removal efficiency of Ni‐COF‐E to below 20%. Addition of silver nitrate (AgNO_3_, e^−^ scavenger) completely quenched the photocatalytic activity. We quantitatively analyzed the suppression efficiencies of the *p*‐BQ and AgNO_3_ scavengers (Table S6). Relative to the blank control, the inhibition efficiencies after addition of *p*‐BQ and AgNO_3_ were 81.54% and 95.93%, respectively. Consequently, the •O_2_^−^‐mediated pathway accounts for 85.00% of the photocatalytic reaction, while a minor fraction of the photogenerated electrons (15.00%) may directly participate in the reduction of U(VI) without proceeding through the •O_2_^−^ pathway. These results demonstrated that the •O_2_^−^‐mediated pathway as the dominant mechanism.

Direct evidence for •O_2_^−^ generation was obtained by electron paramagnetic resonance (EPR) spectroscopy using DMPO as the spin trap. Under light irradiation for 4 min, Ni‐COF‐E exhibited intense characteristic signals of the DMPO‐•O_2_^−^ adduct, while no signal was observed in the dark (Figure 6b; Figure S18). The production of •O_2_^−^ was further quantified by the nitro blue tetrazolium (NBT) method (Figures S19 and S20), which monitored the absorbance decay of NBT upon selective reaction with •O_2_^−^ [53, 54]. As shown in Figure 6c–e, the time‐resolved absorbance decay of NBT in the presence of Ni‐COF‐E was rapid under light irradiation, reflecting efficient •O_2_^−^ generation. The corresponding •O_2_^−^ generation rate (Figure 6f) was 8.48 µM h^−1^ for Ni‐COF‐E, substantially outperforming Ni‐COF‐B (4.74 µM h^−1^) and Ni‐COF‐F (0.89 µM h^−1^). This superior •O_2_^−^ yield is fully consistent with the Zenith Ni sites achieved through ligand engineering.

Theoretical insights into O_2_ activation were obtained from DFT calculations. The O_2_ adsorption energy (Δ*E*_ads_) on the Ni site of Ni‐COF‐E was −3.26 eV, significantly more negative than those on Ni‐COF‐B (−2.16 eV) and Ni‐COF‐F (−1.05 eV) (Figure 6g). This highly favorable adsorption energy indicated that Ni‐COF‐E possessed the strongest affinity for O_2_ among the three Ni‐COFs, a direct consequence of the Zenith electronic state. The free energy diagrams for the oxygen reduction pathway (O_2_ → *O_2_ → *OOH) further corroborate this conclusion (Figure 6h). Ni‐COF‐E exhibited the lowest kinetic barrier for the formation of the key *OOH intermediate, which was the rate‐determining step for •O_2_^−^ generation. In contrast, Ni‐COF‐B and Ni‐COF‐F displayed substantially higher energy barriers, consistent with their lower •O_2_^−^ yields.

Based on the combined experimental and theoretical results, Figure 6i illustrates the photocatalytic •O_2_^−^ generation over Ni‐COF‐E. The Ni site in Ni‐COF‐E, featuring the highest electron density within the present series (the Zenith state), serves as the core for O_2_ adsorption and activation, preferentially capturing dissolved O_2_ to form the *O_2_ intermediate. Subsequently, *O_2_ captured a photogenerated electron and coupled with a proton (H^+^) from the solution to convert into the key *OOH intermediate. *OOH then underwent further reductive transformation to produce •O_2_^−^. The as‐generated •O_2_^−^ radicals diffused into the solution and efficiently reduced U(VI) to U(IV). This pathway clearly revealed how the Zenith Ni site lowers the O_2_ activation barrier and promoted •O_2_^−^ generation, thereby driving efficient photocatalytic uranium reduction.

## 3 Conclusions

In summary, we demonstrated a direct ligand engineering approach to finely control the local electronic state of single Ni sites in COFs. By screening the coordinated diketone units, we identified a peak optimum within the present series that we define as the Zenith electronic state, realized in the acenaphthylene‐1,2‐dione‐derived Ni‐COF‐E. This optimum electron density simultaneously promotes strong O_2_ binding and reduces the energy barrier for •O_2_^−^ formation, resulting in an outstanding •O_2_^−^ generation rate of 8.48 µM h^−1^. Accordingly, Ni‐COF‐E exhibits superior photocatalytic U(VI) reduction performance, achieving 95.3 % removal in simulated wastewater, exceeding 99 % in real uranium wastewater, and a maximum extraction capacity of 818.76 mg g^−1^. This work highlights direct ligand engineering as a powerful route to tailor single‐metal sites in COFs and positions the Zenith electronic state as a new design principle for •O_2_^−^‐mediated photocatalysis with potential beyond uranium extraction.

## Author Contributions

**Guihong Wu**: Investigation, methodology, validation and Writing – original draft. **Fengtao Yu**: Writing – review and editing, funding acquisition, project administration. **Lin Wen**: Methodology and investigation. **Sai‐Jin Xiao**: Investigation and validation. **Fangru Song**: Investigation, formal analysis and validation. **Jian‐Ding Qiu**: Data curation, investigation and methodology.

## Conflicts of Interest

The authors declare no conflict of interest.

## Supporting information




**Supporting File**: advs77949‐sup‐0001‐SuppMat.docx.

## Data Availability

The data that support the findings of this study are available from the corresponding author upon reasonable request.
