# Supplementary material for: Unlocking Zenith Electronic State of Ni Sites in Ligand‐Engineered COFs for Efficient •O2 −‐Driven Uranium Photoreduction
Source: Adv Sci (Weinh). 2026 Sep 27:e77949. Online ahead of print. doi: 10.1002/advs.77949 (PMC13616295; doi:10.1002/advs.77949)
Supplement: Supplementary file 1 [file ADVS-9999-e77949-s001.docx]

**Supplementary Information**

**Unlocking Zenith Electronic State of Ni Sites in Ligand-Engineered COFs for Efficient •O_2_⁻-Driven Uranium Photoreduction**

Guihong Wu, Fengtao Yu*, Lin Wen, Sai-Jin Xiao, Fangru Song, Jian-Ding Qiu*

Dr. G. Wu, Prof. F. Yu, Dr. L. Wen, Prof. S. Xiao, Dr. F. Song, Prof. J. Qiu

*National Key Laboratory of Uranium Resources Exploration-Mining and Nuclear Remote Sensing, East China University of Technology, Nanchang, 330013, China.*

E-mail addresses: [fty853815622@ecut.edu.cn](mailto:fty853815622@ecut.edu.cn); [jdqiu@ecut.edu.cn](mailto:jdqiu@ecut.edu.cn)

*Corresponding authors

**Table of Contents**

[**Part 1. Materials and methods**	3](#_Toc232694283)

[**Part 2. Experimental Section**	6](#_Toc232694284)

[**Part 3. Synthesis**	7](#_Toc232694285)

[**Part 4. Supplemental Figures**	9](#_Toc232694286)

[**Part 5. Supplemental Tables**	17](#_Toc232694287)

[**References**	23](#_Toc232694288)

Part 1. Materials and methods

**Materials**

Acetic acid, 1,3,5-tris(4-aminophenyl)benzene (TPB), uranyl nitrate hexahydrate, benzil, acenaphthenequinone, phenanthrenequinone and arsenazo Ⅲ were purchased from Alfa Aesar Ltd., Tetrahydrofuran (THF), 1,4-dioxane, methanol, mesitylene, 4-hydroxy-2,2,6,6-tetramethyl-piperidinooxy (TEMPO), 5,5-Dimethyl-1-pyrroline-N-oxide (DMPO), tertiary butanol (TBA), nickel chloride hexahydrate (NiCl_2_·6H_2_O), silver nitrate (AgNO_3_), and 1,4-benzoquinone (*p*-BQ) were purchased from Sinopharm Chemical Reagent Co., Ltd. Ultrapure water was prepared from the Millipore system (18.25 MΩ-cm). All the electrochemical measurement used 0.1 M Na_2_SO_4_. All reagents were used as received unless otherwise indicated.

**Characterization methods**

FT-IR spectra were calendared with a Bruker TENSOR 27 instrument. UV-vis diffuse reflection spectrum (DRS) was received with UV-vis spectrophotometer (UV-2550, Shimadzu, Japan). Powder X-ray diffractions (PXRD) data were collected on a Bruker AXS D8 Advance A25 powder X-ray diffractometer (40 kV, 40 mA) using Cu Kα radiation (λ = 1.5406 Å). X-ray photoelectron spectroscopy (XPS) spectra were gathered with a Thermo VG Multilab 2000X with Al Kα irradiation. N_2_ adsorption and desorption isotherms were carried out at 77 K with a micromeritics ASAP 2020 M system. The samples were outgassed for 12 h at 120°C under vacuum prior to the gas adsorption studies. The surface areas were evaluated using Brunauer-Emmett-Teller (BET) model applied between P/P_0_ values of 0.05 and 0.20. The pore size distributions were calculated using the non-localized density functional theory (NLDFT) method. Field-emission scanning electron microscope (FE-SEM) images were taken by Nova Nanosem 450. Energy-dispersive X-ray spectroscopy (EDS) were conducted using SEM equipped with an EDX detector. Metal ions concentrations were determined using an iCAP Q inductively coupled plasma mass spectrometry (ICP-MS, Thermo Fisher Scientific, USA). Electron spin resonance (ESR) was used to investigate the reactive species, which was recorded using a Bruker A300 spectrometer. Specifically, the 5,5-dimethyl-1-pyrroline-N-oxide (DMPO) was used to trap the superoxide radical (•O_2_^-^) and hydroxyl radicals (•OH). As well, 4-hydroxy-2,2,6,6-tetramethyl-piperidinooxy (TEMPO) was a typical electron capture agent, which would be reduced to produce an ESR-silent product (TEMPOH). Photocurrent responses (i-t) and Electrochemical impedance spectroscopy (EIS) was recorded on CHI660 electrochemical workstation in a classical three-electrode configuration consisting of a modified working electrode, a platinum counter electrode, and an Ag/AgCl reference electrode.

**DFT calculation methods**

The all geometries, including Ni-COF-B, Ni-COF-F, Ni-COF-E, and corresponding structures by adsorbing UO_2_^2+^, were optimized at the CAM-B3LYP/6-31G(d) level of theory using Gaussian 16. Vibrational frequency analyses were performed to confirm that the optimized structures corresponded to an energy minimum. Based on the energy with the zero-point correction, all isomers were selected for CAM-B3LYP/6-31++G (d, p) single point energy calculations. Hence, the all energy for calculating the adsorption energy were corrected by the zero-point energy. The adsorption energy (*E*_ads_) was calculated as follows: *E*_ads_ = -(*E*_total_ – *E*_substrate_ – *E*_gas-phase_ _adsorbate_), where *E*_total_ was the total energy of the adsorption system, *E*_substrate_ was the energy of the clean substrate, and *E*_gas-phase_ adsorbate was the energy of the gas-phase molecule.

**Computational details**

All geometric optimization and total energy calculations in this study were performed at the spin-polarized DFT by using the Vienna Ab initio Simulation Package (VASP) [20-23]. The projector augmented wave (PAW) method [24], and the Perdew-Burke-Ernzerh (PBE) [25] functional under the generalized gradient approximation (GGA) were applied throughout the calculations, and a cut-off energy of 400 eV was used. To avoid interactions between neighboring slabs, a vacuum layer of ~15 Å was set. We constructed altogether four COF surfaces, and each surface model is kept relaxed. The Brillouin zones were sampled with *k* points of 3×3×1 Gamma grids for different COF system calculations. The convergence criteria of energy and force in the calculations were set as 1.0 × 10^-4^ eV and 0.05 eV/Å, respectively.

The charge density difference is one of the important methods for studying electronic structures. It provides an intuitive view of the electron flow after the interaction between two fragments, reveals the changes in electron density during molecular formation, and helps explore the nature of chemical bonds. The charge density difference is expressed as: The total charge density of the system minus the charge densities of its constituent fragments (which can involve two or more fragments).

Part 2. Experimental Section

**Photocatalytic uranium extraction**

The experiments were conducted based on the photocatalytic reduction methods documented in the literature. We chose a certain concentration of uranium (VI) solution for the experiment. The entire unit is placed in circulating condensed water to maintain the system temperature at room temperature. Simply disperse 5.0 mg of the COFs photocatalyst in 25 mL of the U(VI) solution and ultrasonically disperse it uniformly. The suspension is subsequently transferred to the reactor (CEL-APR250H), where it is stirred in a dark environment for 60 mins to reach adsorption equilibrium. Then the reactor was irradiated with 300 W Xenon lamp (SHX-F300) light source, and the visible light was kept in the range of (420 ≤ λ ≤ 780 nm) by long-pass cut-off filter. During the reaction, 2.0 mL of solution was extracted and filtered at a given time to obtain a photocatalyst-free supernatant. Noticeable, that residual concentrations of uranium and other interfering metal ions were determined using ICP-MS.

**Uranium extraction from real wastewater**

The actual wastewater, which was taken from a uranium company in China, utilized in the experiment has undergone filtration to eliminate the insoluble solids for subsequent reactions. 5.0 mg of COFs powder was added to 25 mL of the actual wastewater, and the ultrasonic dispersion was made uniform and stirred under the irradiation of a 300W Xenon lamp. Small and equal samples of the dispersion were extracted periodically and filtered for the analysis of U (VI) concentration.

**Recyclability test**

After one run of adsorption, the adsorbent was regenerated by treatment with the elution solution of HNO_3_ (0.1 M) solution and reused for another adsorption experiment. For 5.0 mg adsorbents, 50 mL elution solution was used to elute the binding uranium for 30 min at RT. The elution efficiency (E, %) was determined by using Equation: $E=\frac{C_{e}\times V_{e}}{(C_{0}-C_{e})\times V_{t}}\times$100%, where C_e_ (mg L^-1^) is the uranium concentration in elution solution, V_e_ (L) is the volume elution solution, C_t_ (mg L^-1^) is the uranium concentration in uranium-containing wastewater after uranium adsorption, C_0_ (mg L^-1^) is the initial uranium concentration of uranium-containing wastewater, V_t_ (L) is the volume of uranium-containing wastewater used for adsorption. The resulting suspension was filtered and washed with ultra-pure water till the supernatant became neutral. After being dried under vacuum, the resultant material was used for another adsorption experiment.

Part 3. Synthesis

**Synthesis of Ni-COF-B**

TPB (0.067 mmol, 23.5 mg), benzil (0.1 mmol, 21.0 mg), and NiCl_2_·6H_2_O (0.1 mmol, 23.8 mg) were added to an ampoule. Subsequently, 1.5 mL of mesitylene, 1.5 mL of ethanol, and 0.1 mL of 6.0 M acetic acid were added sequentially to the tube, and the mixture was ultrasonicated for 10 min. The resulting mixture was frozen using liquid nitrogen, degassed for 20 min, and flame-sealed under vacuum. The sealed tube was heated at 120°C for 3 days. The solid was collected by centrifugation, washed twice each with 1,4-dioxane, tetrahydrofuran, and ethanol, and then dried under vacuum to obtain the powder.

**Synthesis of Ni-COF-F**

TPB (0.067 mmol, 23.5 mg), phenanthrene-9,10-dione (0.1 mmol, 18.2 mg), and NiCl_2_·6H_2_O (0.1 mmol, 23.8 mg) were added to an ampoule. Subsequently, 2.1 mL of mesitylene, 0.9 mL of ethanol, and 0.1 mL of 6.0 M acetic acid were added sequentially to the tube, and the mixture was ultrasonicated for 10 min. The resulting mixture was frozen using liquid nitrogen, degassed for 20 min, and flame-sealed under vacuum. The sealed tube was heated at 120°C for 3 days. The solid was collected by centrifugation, washed twice each with 1,4-dioxane, tetrahydrofuran, and ethanol, and then dried under vacuum to obtain the powder.

**Synthesis of Ni-COF-E**

TPB (0.067 mmol, 23.5 mg), acenaphthylene-1,2-dione (0.1 mmol, 20.8 mg), and NiCl_2_·6H_2_O (0.1 mmol, 23.8 mg) were added to an ampoule. Subsequently, 1.5 mL of mesitylene, 1.5 mL of ethanol, and 0.1 mL of 6.0 M acetic acid were added sequentially to the tube, and the mixture was ultrasonicated for 10 min. The resulting mixture was frozen using liquid nitrogen, degassed for 20 min, and flame-sealed under vacuum. The sealed tube was heated at 120°C for 3 days. The solid was collected by centrifugation, washed twice each with 1,4-dioxane, tetrahydrofuran, and ethanol, and then dried under vacuum to obtain the powder.

**Part 4. Supplemental Figures**

**Figure S1**. ABC stacked structure top and side views of Ni-COFs.

**Figure S2.** FT-IR spectra of Ni-COF-B.

**Figure S3.** FT-IR spectra of Ni-COF-F.

**Figure S4.** SEM images for Ni-COF-B and the corresponding EDX mappings.

**Figure S5.** SEM images for Ni-COF-E and the corresponding EDX mappings.

**Figure S6.** SEM images for Ni-COF-F and the corresponding EDX mappings.

**Figure S7.** The linear relationship between the test results of Ni^2+^ standard solutions of different concentrations and ICP-MS.

**Figure S8.** Nitrogen adsorption/desorption isotherms of Ni-COF-B.

**Figure S9.** Nitrogen adsorption/desorption isotherms of Ni-COF-E.

**Figure S10.** Nitrogen adsorption/desorption isotherms of Ni-COF-F.

**Figure S11.** Theoretical Pore Widths of (a) Ni-COF-B, (b) Ni-COF-E and (c) Ni-COF-F.

**Figure S12.** Thermogravimetric curves of Ni-COFs.

**Figure S13.** Mott-Schottky plots for Ni-COFs.

**Figure S14.** HOMO and LUMO distributions of the simplified TTP, BTB and NTN.

**Figure S15**. PXRD of Ni-COF-E after five cycles.

**Figure S16**. FT-IR spectra of Ni-COF-E after five cycles.

**Figure S17.** FTIR spectra of Ni-COF-E and Ni-COF-E-U.

**Figure S18.** EPR spectra of TEMPO-e^-^.

**Figure S19.** Reactions for detecting •O_2_^-^ with a coloring reagent of Nitro Blue Tetrazolium Chloride monohydrate (NBT).

**Figure S20.** (a) UV−vis absorption spectra of NBT solutions with different concentrations. (b) The linear relationship between NBT concentration and the absorbance intensity at the absorbance peak of 259 nm.

**Part 5. Supplemental Tables**

**Table S1**. ICP-MS of Ni-COFs

| **Sample** | **Element** | **Theoretical content** | **Experimental content** |
| --- | --- | --- | --- |
| Ni-COF-B | Ni | 12.58% | 12.23% |
| Ni-COF-E | Ni | 13.38% | 11.22% |
| Ni-COF-F | Ni | 12.63% | 11.53% |

**Table S2**. Fractional atomic coordinates for the unit cell of Ni-COF-B with ABC stacking.

| Sample name: Ni-COF-B | | | | | | | |
| --- | --- | --- | --- | --- | --- | --- | --- |
| Space group: R 3 (146)-trigonal | | | | | | | |
| *a* = *b* = 28.156 Å, *c* = 11.087 Å, *α* = *β* = 90°, *γ* = 120° | | | | | | | |
| *R*_wp_ = 5.41%, *R*_p_ = 7.45% | | | | | | | |
| C1 | -0.31563 | -0.17915 | 0.30208 | C29 | -0.64821 | -0.27706 | 0.53946 |
| C2 | -0.37189 | -0.20634 | 0.34805 | C30 | -0.5508 | -0.25592 | 0.52077 |
| C3 | -0.40982 | 0.73388 | 0.33499 | C31 | -0.53366 | -0.217 | 0.42723 |
| C4 | -0.40055 | 0.69648 | 0.39987 | C32 | -0.47832 | -0.18691 | 0.39479 |
| C5 | -0.3175 | 0.76249 | 0.12975 | C33 | -0.43931 | -0.19481 | 0.45619 |
| C6 | -0.2932 | -0.21 | 0.23783 | C34 | -0.45578 | -0.23067 | 0.55515 |
| C7 | -0.45757 | 0.71458 | 0.26518 | C35 | -0.51115 | -0.26124 | 0.5869 |
| C8 | -0.49613 | 0.65866 | 0.26242 | H36 | -0.46534 | 0.74295 | 0.21477 |
| C9 | -0.48701 | 0.62158 | 0.32795 | H37 | -0.53324 | 0.64423 | 0.21019 |
| C10 | -0.43931 | 0.64051 | 0.39669 | H38 | -0.51706 | 0.57828 | 0.32627 |
| C11 | -0.29513 | 0.73549 | 0.06591 | H39 | -0.43296 | 0.61187 | 0.44931 |
| C12 | -0.24822 | 0.73608 | 0.10904 | H40 | -0.31383 | 0.71451 | -0.0175 |
| C13 | -0.22452 | -0.23774 | 0.21785 | H41 | -0.23033 | 0.71612 | 0.05827 |
| C14 | -0.24718 | -0.21128 | 0.28268 | H42 | -0.18851 | -0.23779 | 0.2518 |
| N15 | -0.28736 | -0.12654 | 0.32305 | H43 | -0.2285 | -0.19123 | 0.36678 |
| Ni16 | -0.32917 | -0.1015 | 0.40362 | H44 | -0.23751 | -0.1042 | 0.10498 |
| N17 | -0.38555 | -0.17448 | 0.40489 | H45 | -0.13795 | -0.0597 | 0.06788 |
| Cl18 | -0.37715 | -0.07287 | 0.49881 | H46 | -0.10787 | -0.0314 | 0.44937 |
| Cl19 | -0.26492 | -0.01639 | 0.39753 | H47 | -0.20728 | -0.07731 | 0.48654 |
| C20 | -0.22945 | -0.09384 | 0.29861 | H48 | -0.03666 | 0.06395 | 0.24214 |
| C21 | -0.20931 | -0.08765 | 0.18016 | H49 | -0.63388 | -0.23353 | 0.53384 |
| C22 | -0.15281 | -0.0625 | 0.15907 | H50 | -0.56344 | -0.21259 | 0.37379 |
| C23 | -0.11595 | -0.0425 | 0.25599 | H51 | -0.4663 | -0.16015 | 0.31692 |
| C24 | -0.13592 | -0.04653 | 0.37363 | H52 | -0.42608 | -0.23748 | 0.6024 |
| C25 | -0.19233 | -0.07239 | 0.39482 | H53 | -0.52333 | -0.29078 | 0.65963 |
| C26 | -0.05689 | -0.0207 | 0.23699 | H54 | -0.36427 | 0.71081 | 0.45542 |
| C27 | -0.02067 | 0.03607 | 0.23654 | H55 | -0.35317 | 0.76274 | 0.09402 |
| C28 | -0.60998 | -0.29521 | 0.53879 |  |  |  |  |

**Table S3**. Fractional atomic coordinates for the unit cell of Ni-COF-E with ABC stacking.

| Sample name: Ni-COF-E | | | | | | | |
| --- | --- | --- | --- | --- | --- | --- | --- |
| Space group: R 3 (146)-trigonal | | | | | | | |
| *a* = *b* = 28.756 Å, *c* = 11.087 Å, *α* = *β* = 90°, *γ* = 120° | | | | | | | |
| *R*_wp_ = 3.20%, *R*_p_ = 4.22% | | | | | | | |
| C1 | -0.30483 | -0.23739 | 0.33277 | C26 | -0.55431 | -0.29977 | 0.32524 |
| C2 | -0.36183 | -0.26589 | 0.33497 | C27 | -0.52116 | -0.26576 | 0.2338 |
| C3 | -0.37975 | 0.67665 | 0.33289 | C28 | -0.46606 | -0.24719 | 0.23535 |
| C4 | -0.28691 | -0.27693 | 0.33485 | C29 | -0.44322 | -0.26141 | 0.32969 |
| C5 | -0.42994 | 0.63001 | 0.33009 | C30 | -0.47654 | -0.2963 | 0.42006 |
| C6 | -0.43123 | 0.58021 | 0.32933 | C31 | -0.53189 | -0.31569 | 0.41738 |
| C7 | -0.3832 | 0.57775 | 0.33144 | C32 | 1.3403 | 1 | 1.00054 |
| C8 | -0.28347 | 0.62762 | 0.3363 | C33 | 0.70788 | 0.70788 | 1.00053 |
| C9 | -0.23544 | -0.3219 | 0.3384 | H34 | -0.46805 | 0.63291 | 0.3285 |
| C10 | -0.23673 | -0.27339 | 0.33765 | H35 | -0.47138 | 0.5411 | 0.32699 |
| N11 | -0.28068 | -0.18565 | 0.33116 | H36 | -0.38517 | 0.53716 | 0.33116 |
| Ni12 | -0.33333 | -0.16446 | 0.33388 | H37 | -0.2815 | 0.589 | 0.33657 |
| N13 | -0.38598 | -0.2383 | 0.33659 | H38 | -0.19486 | -0.31994 | 0.34076 |
| Cl14 | -0.39207 | -0.13868 | 0.34508 | H39 | -0.19763 | -0.23323 | 0.33931 |
| Cl15 | -0.27459 | -0.07994 | 0.32268 | H40 | -0.20812 | -0.18375 | 0.17165 |
| C16 | -0.22344 | -0.15152 | 0.33806 | H41 | -0.10778 | -0.11897 | 0.17748 |
| C17 | -0.19013 | -0.15309 | 0.24769 | H42 | -0.12753 | -0.04733 | 0.5101 |
| C18 | -0.13477 | -0.11713 | 0.25038 | H43 | -0.22727 | -0.11423 | 0.50822 |
| C19 | -0.11235 | -0.07879 | 0.34252 | H44 | -0.06961 | 0.02964 | 0.34111 |
| C20 | -0.1455 | -0.07793 | 0.43396 | H45 | -0.59706 | -0.23408 | 0.32666 |
| C21 | -0.20061 | -0.11446 | 0.43241 | H46 | -0.53914 | -0.25313 | 0.15765 |
| C22 | -0.05489 | -0.03861 | 0.34144 | H47 | -0.4394 | -0.22029 | 0.15953 |
| C23 | -0.03841 | 0.01623 | 0.34132 | H48 | -0.45854 | -0.30896 | 0.4961 |
| C24 | -0.61178 | -0.31705 | 0.32632 | H49 | -0.55889 | -0.34452 | 0.49028 |
| C25 | -0.62826 | -0.27869 | 0.32645 |  |  |  |  |

**Table S4**. Fractional atomic coordinates for the unit cell of Ni-COF-F with ABC stacking.

| Sample name: Ni-COF-F | | | | | | | |
| --- | --- | --- | --- | --- | --- | --- | --- |
| Space group: R 3 (146)-trigonal  *a* = *b* = 28.756 Å, *c* = 11.087 Å, *α* = *β* = 90°, *γ* = 120° | | | | | | | |
| *R*_wp_ = 4.41%, *R*_p_ = 5.57% | | | | | | | |
| C1 | -0.30555 | -0.2554 | 0.35103 | C28 | -0.6112 | -0.31769 | 0.29064 |
| C2 | -0.36313 | -0.28041 | 0.32253 | C29 | -0.6271 | -0.27823 | 0.29094 |
| C3 | -0.39045 | 0.66281 | 0.27742 | C30 | -0.55319 | -0.3012 | 0.29698 |
| C4 | -0.36454 | 0.63135 | 0.28852 | C31 | -0.5141 | -0.25531 | 0.24031 |
| C5 | -0.31131 | 0.65378 | 0.3452 | C32 | -0.45895 | -0.23913 | 0.24915 |
| C6 | -0.28198 | -0.29078 | 0.37552 | C33 | -0.44152 | -0.26686 | 0.31799 |
| C7 | -0.4392 | 0.64072 | 0.21816 | C34 | -0.481 | -0.3128 | 0.37441 |
| C8 | -0.46356 | 0.58815 | 0.17535 | C35 | -0.53629 | -0.33034 | 0.3629 |
| C9 | -0.43887 | 0.55676 | 0.18965 | H36 | -0.45968 | 0.66552 | 0.20411 |
| C10 | -0.38956 | 0.57834 | 0.24552 | H37 | -0.5041 | 0.5699 | 0.12789 |
| C11 | -0.29037 | 0.61958 | 0.37157 | H38 | -0.45968 | 0.51366 | 0.15506 |
| C12 | -0.2419 | 0.63939 | 0.43008 | H39 | -0.36931 | 0.55317 | 0.25661 |
| C13 | -0.21416 | -0.3065 | 0.46424 | H40 | -0.3137 | 0.57546 | 0.34431 |
| C14 | -0.23448 | -0.27215 | 0.43817 | H41 | -0.22495 | 0.61186 | 0.45001 |
| N15 | -0.28108 | -0.20296 | 0.36335 | H42 | -0.17429 | -0.28951 | 0.51418 |
| Ni16 | -0.32896 | -0.17771 | 0.37566 | H43 | -0.21167 | -0.22848 | 0.46889 |
| N17 | -0.38412 | -0.24844 | 0.3319 | H44 | -0.19813 | -0.2139 | 0.26819 |
| Cl18 | -0.36922 | -0.16658 | 0.44382 | H45 | -0.09964 | -0.14232 | 0.26922 |
| Cl19 | -0.26525 | -0.08 | 0.32277 | H46 | -0.13975 | -0.03143 | 0.44876 |
| C20 | -0.22385 | -0.16424 | 0.3661 | H47 | -0.23802 | -0.1042 | 0.45274 |
| C21 | -0.18551 | -0.17348 | 0.31226 | H48 | -0.07208 | 0.02716 | 0.3592 |
| C22 | -0.13058 | -0.1334 | 0.31092 | H49 | -0.59542 | -0.23378 | 0.29144 |
| C23 | -0.11336 | -0.0827 | 0.36007 | H50 | -0.52603 | -0.22952 | 0.18487 |
| C24 | -0.15207 | -0.07267 | 0.40965 | H51 | -0.42784 | -0.20215 | 0.19813 |
| C25 | -0.20687 | -0.11314 | 0.4123 | H52 | -0.46931 | -0.33799 | 0.43275 |
| C26 | -0.05536 | -0.04037 | 0.35908 | H53 | -0.56732 | -0.36905 | 0.40867 |
| C27 | -0.04004 | 0.01487 | 0.35912 |  |  |  |  |

**Table S5**. Composition and concentration of ions in uranium mine wastewater which is taken from a uranium company in China.

| **Composition of Sample** | **Concentration** |
| --- | --- |
| U | 5.8 mg L^-1^ |
| Th | 2.1 mg L^-1^ |
| As | 1.3 mg L^-1^ |
| Mn | 318 mg L^-1^ |
| Ca | 217 mg L^-1^ |
| Al | 85 mg L^-1^ |
| F | 304 mg L^-1^ |
| Ra | 29 BqL^-1^ |
| SO_4_^2-^ | 1011 mg L^-1^ |
| COD | 98 mg L^-1^ |

**Table S6**. The inhibition rate relative to the blank group after adding the sacrificial agents *p*-BQ and AgNO_3_, as well as the ratio of the •O_2_⁻ pathway and the direct electron transfer pathway.

| **Sacrificial agent:** | **Blank** | ***p*-BQ** | **AgNO_3_** |
| --- | --- | --- | --- |
| **Removal rate (%)** | 95.30 | 17.59 | 3.88 |
| **Inhibition rate relative to blank group (%)** | \ | 81.54 | 95.93 |
| **Ratio of the •O_2_⁻ pathway (%)** | \ | 85.00 | \ |
| **Ratio of the direct e⁻ transfer pathway (%)** | \ | \ | 15.00 |

**References**

1. J. Liu, M. Lin, J. Huang, et al., "*sp^2^*-c Linked Cu-Based Metal-Covalent Organic Framework for Chemical and Photocatalysis Synergistic Reduction of Uranium," *Chemical Engineering Journal* 491 (2024): 151982, <https://doi.org/10.1016/j.cej.2024.151982>.

2. M. Hao, M. Lei, J. Zhang, et al., "Flexible Units in Covalent Organic Frameworks Promote Photocatalytic Uranium Extraction from Wastewater," *CCS Chemistry* 0 (2026): 1-14, <https://doi.org/10.31635/ccschem.025.202506940>.

3. X. X. Wang, C.-R. Zhang, R.-X. Bi, et al., "Tuning Local Charge Polarization of Covalent Organic Frameworks for Enhanced Photocatalytic Uranium (VI) Reduction," *Chemical Engineering Journal* 499 (2024): 156483, <https://doi.org/10.1016/j.cej.2024.156483>.

4. Y. Ma, C. Gan, L. Liu, et al., "Polarization-Π Strategy for Modulating Excited-State Band Structures of Covalent Organic Frameworks to Enhance Photocatalytic Uranium Extraction," *Advanced Functional Materials* 36 (2026): e23125, <https://doi.org/10.1002/adfm.202523125>.

5. H.-X. He, C.-R. Zhang, X.-J. Chen, R.-P. Liang, J.-D. Qiu, "Reconstruction of Built-in Electric Field in Covalent Organic Frameworks through Defect Engineering for Photocatalytic Reduction of Uranium," *ACS Applied Polymer Materials* 7 (2025): 4525-4534, <https://doi.org/10.1021/acsapm.5c00178>.

6. Y. Chen, S. Dong, Y. Zhao, et al., "Molecular-Level Engineering of Amino Functional Group Distribution in Porous Organic Polymers for High-Performance Photocatalytic Uranium Removal," *Separation and Purification Technology* 380 (2026): 135166, <https://doi.org/10.1016/j.seppur.2025.135166>.

7. X. Zhong, Q. Ling, Z. Ren, B. Hu, "Immobilization of U(VI) onto Covalent Organic Frameworks with the Different Periodic Structure by Photocatalytic Reduction," *Applied Catalysis B: Environmental* 326 (2023): 122398, <https://doi.org/10.1016/j.apcatb.2023.122398>.

8. X. Wang, W. Xu, W.-R. Cui, "Modulating Conjugated Microporous Polymers Via Cyclization as a Remarkable Photo-Enhanced Uranium Recovery Platform," *Journal of Hazardous Materials* 463 (2024): 132846, <https://doi.org/10.1016/j.jhazmat.2023.132846>.

9. H. Zhang, W. Liu, A. Li, et al., "Three Mechanisms in One Material: Uranium Capture by a Polyoxometalate–Organic Framework through Combined Complexation, Chemical Reduction, and Photocatalytic Reduction," *Angewandte Chemie International Edition* 58 (2019): 16110-16114, <https://doi.org/10.1002/anie.201909718>.

10. F. Yu, F. Song, R. Wang, M. Xu, F. Luo, "Sulfonated Perylene-Based Conjugated Microporous Polymer as a High-Performance Adsorbent for Photo-Enhanced Uranium Extraction from Seawater," *Polymer Chemistry* 12 (2021): 867-875, <https://doi.org/10.1039/D0PY01656J>.

11. Y. Li, X. Guo, X. Li, et al., "Redox-Active Two-Dimensional Covalent Organic Frameworks (COFs) for Selective Reductive Separation of Valence-Variable, Redox-Sensitive and Long-Lived Radionuclides," *Angewandte Chemie International Edition* 59 (2020): 4168-4175, <https://doi.org/10.1002/anie.201916360>.

12. Y. Zhao, S. Li, G. Fu, et al., "Construction of Layer-Blocked Covalent Organic Framework Heterogenous Films Via Surface-Initiated Polycondensations with Strongly Enhanced Photocatalytic Properties," *ACS Central Science* 10 (2024): 775-781, <https://doi.org/10.1021/acscentsci.3c01195>.

13. R. Wen, Y. Li, M. Zhang, et al., "Graphene-Synergized 2d Covalent Organic Framework for Adsorption: A Mutual Promotion Strategy to Achieve Stabilization and Functionalization Simultaneously," *Journal of Hazardous Materials* 358 (2018): 273-285, <https://doi.org/10.1016/j.jhazmat.2018.06.059>.

14. C. R. Zhang, W. R. Cui, C. P. Niu, et al., "rGO-Based Covalent Organic Framework Hydrogel for Synergistically Enhance Uranium Capture Capacity through Photothermal Desalination," *Chemical Engineering Journal* 428 (2022): 131178, <https://doi.org/10.1016/j.cej.2021.131178>.

15. S. J. Xiao, J. Huang, A. T. Qiu, et al., "Advanced “Turn-on” Colorimetric Uranium Platform Based on the Enhanced Nanozyme Activity of a Donor-Acceptor Structured Covalent Organic Framework," *Analytica Chimica Acta* 1302 (2024): 342503, <https://doi.org/10.1016/j.aca.2024.342503>.

16. R. Zhang, L. Tao, C. P. Niu, et al., "Phosphorylated Covalent Organic Framework/Graphene Composites for Photoelectrothermal Integrated Collaborative Reduction of Uranium," *Chemical Engineering Journal* 488 (2024): 151007, <https://doi.org/10.1016/j.cej.2024.151007>.

17. X. J. Chen, C.-R. Zhang, Y. J. Cai, et al., "Construction of a Bifunctional Redox-Site Conjugated Covalent–Organic Framework for Photoinduced Precision Trapping of Uranyl Ions," *Inorganic Chemistry* 63 (2024): 11459-11469, <https://doi.org/10.1021/acs.inorgchem.4c01649>.

18. W. R. Cui, W. Xu, W. B. Qiu, "Constructing an Ultra-stable Imidazole Covalent Organic Framework for Concurrent Uranium Detection and Recovery," *Ecotoxicology and Environmental Safety* 252 (2023): 114639, <https://doi.org/10.1016/j.ecoenv.2023.114639>.

19. R. X. Bi, X. Liu, Z. H. Peng, et al., "Covalent Bonding Confining Polyoxometalates in Covalent Organic Frameworks for Efficiently Capturing Uranium," *Separation and Purification Technology* 330 (2024): 125333, <https://doi.org/10.1016/j.seppur.2023.125333>.

20. G. Kresse, J. Furthmüller, "Efficiency of Ab-Initio Total Energy Calculations for Metals and Semiconductors Using a Plane-Wave Basis Set," *Computational Materials Science* 6 (1996): 15-50, <https://doi.org/10.1016/0927-0256(96)00008-0>.

21. G. Kresse, J. Hafner, "Ab Initio Molecular-Dynamics Simulation of the Liquid-Metal--Amorphous-Semiconductor Transition in Germanium," *Physical Review B* 49 (1994): 14251-14269, <https://doi.org/10.1103/PhysRevB.49.14251>.

22. G. Kresse, J. Hafner, "Ab Initio Molecular Dynamics for Liquid Metals," *Physical Review B* 47 (1993): 558-561, <https://doi.org/10.1103/PhysRevB.47.558>.

23. G. Kresse, J. Furthmüller, "Efficient Iterative Schemes for Ab Initio Total-Energy Calculations Using a Plane-Wave Basis Set," *Physical Review B* 54 (1996): 11169-11186, <https://doi.org/10.1103/PhysRevB.54.11169>.

24. P. E. Blöchl, "Projector Augmented-Wave Method," *Physical Review B* 50 (1994): 17953-17979, <https://doi.org/10.1103/PhysRevB.50.17953>.

25. J. P. Perdew, J. A. Chevary, S. H. Vosko, et al., "Atoms, Molecules, Solids, and Surfaces: Applications of the Generalized Gradient Approximation for Exchange and Correlation," *Physical Review B* 46 (1992): 6671-6687, <https://doi.org/10.1103/PhysRevB.46.6671>.
